# Relationship Between Phase Angle Assessed by Bioelectrical Impedance Analysis and Jumping Performance in Rhythmic Gymnasts: An Exploratory Cross-Sectional Study

**DOI:** 10.3390/sports14080315

**Published:** 2026-07-23

**Authors:** Kana Shimizu, Ayumi Yoshikawa, Naoko Yabuno, Yuta Hashimoto, Takuya Nishioka, Shota Yamaguchi, Takayuki Inami

**Affiliations:** 1 Institute of Physical Education, Keio University, 4-1-1, Hiyoshi, Yokohama 223-8521, Japan; nishioka.t@keio.jp (T.N.); yamaguchi.s@keio.jp (S.Y.); inamit@keio.jp (T.I.); 2 Graduate School of Health Management, Keio University, 4-1-1, Hiyoshi, Yokohama 223-8521, Japan; ayumi.y@keio.jp (A.Y.); naoko.yabuno@keio.jp (N.Y.); yuta.hashimoto82310059@keio.jp (Y.H.)

**Keywords:** bioelectrical impedance analysis, jumping performance, rhythmic gymnasts, reactive strength index, countermovement jump

## Abstract

In aesthetic sports, performance is closely linked to body composition; however, quantitative indicators for monitoring athlete conditions remain limited. Bioelectrical impedance analysis-derived phase angle (PhA) reflects cellular health; however, its relationship with performance-related variables in rhythmic gymnasts remains unclear. This study explored the relationships among PhA, body composition, bone density, training time, and jump performance in senior rhythmic gymnasts across competitive levels. Twenty-three female gymnasts (aged 15–22 years) were classified into a top group consisting of athletes who had won major domestic competitions in Japan (*n* = 5) and a general group (*n* = 18). Body composition and PhA were assessed using bioelectrical impedance analysis, jump performance using the countermovement jump and reactive strength index, and bone density using the calcaneal ultrasound method. Although between-group differences were limited, distinct associations between PhA and performance-related variables were observed only in the top group. PhA was positively associated with skeletal muscle mass. In addition, a moderate, non-significant negative association between PhA and countermovement jump performance was observed in the top group. No clear relationships between PhA and body composition or jump performance were observed in the general group. Although these findings should be interpreted cautiously because of the small sample size and exploratory nature of the study, they suggest that the associations between PhA and performance-related variables may differ according to competitive level. Further studies with larger sample sizes and longitudinal designs are needed to clarify the relationships between PhA, performance-related variables, and physiological condition in rhythmic gymnasts.

## 1. Introduction

Rhythmic gymnastics is an aesthetic sport in which scores are influenced not only by flexibility, jumping ability, and precise body control but also by musical expression and the beauty of the body’s lines. Owing to the nature of the sport, athletes are often required to maintain a low body fat percentage—that is, to manage their body composition—while sustaining a high training volume. Rhythmic gymnasts have lower body fat than that of women of the same age in the general population [1], and athletes with higher jumping performance have lower body fat percentages and greater lean body mass [2]. However, excessive energy restriction aimed at maintaining low body fat can lead to low energy availability, resulting in health issues such as menstrual irregularities and decreased bone density [3,4,5]. These risks are particularly likely to manifest in female athletes during adolescence and young adulthood, as the demands of their sport and body image management overlap. These health problems are categorized as the female athlete triad or relative energy deficiency in sports (RED-S) and are frequently observed in female athletes, including those in aesthetic sports [3,4,5]. Therefore, in rhythmic gymnastics, where performance advantages and health risks may coexist due to the demands of body composition management, optimizing athlete condition should consider both physical characteristics that are advantageous for performance and those that pose health risks.

In recent years, bioelectrical impedance analysis (BIA) has been increasingly used as a non-invasive and practical method for assessing physiological condition. BIA measures resistance (R) and reactance (Xc) to an alternating current, from which the phase angle (PhA) is calculated as the arctangent of Xc/R. PhA is a composite indicator reflecting cellular integrity and body fluid distribution and is widely used in clinical settings as an indicator of physiological condition [6,7]. Previous studies in athletic populations have shown that PhA is associated with muscle strength [8], muscle power [9], and muscle function independent of muscle mass [10]. Moreover, PhA is influenced by training load and recovery status [11], with temporary decreases observed following exercise accompanied by muscle damage [12]. These findings highlight its potential as an indicator reflecting physiological parameters not captured by conventional body composition measures and for assessing athletes’ physiological condition, including fatigue accumulation and overtraining [11]. Thus, in aesthetic sports, measuring PhA alongside conventional body composition assessments may provide additional information regarding athletes’ physiological condition.

Although PhA tends to be higher in athletes than in the general population, considerable variability exists across sports and training status [13], and the relationship between PhA and performance-related characteristics in rhythmic gymnastics remains unclear. Evidence from female aesthetic disciplines further highlights this complexity. In ballet, another aesthetic sport, female dancers with conditions, such as leanness and eating disorders, as well as general dancers, show no significant differences in body size or composition; however, PhA values are higher in general dancers [14]. These findings suggest that even among aesthetic athletes with similarly low body weights, cellular health may differ depending on athletes’ training and conditioning status. Although high-level rhythmic gymnasts generally exhibit superior performance (e.g., jumping ability) and greater training volumes, whether these differences are reflected in physiological indicators such as PhA remains unclear. Jumping is a key component of rhythmic gymnastics and contributes substantially to technical execution and overall scoring. Elements such as split leaps and other airborne movements are frequently included in routines and require high levels of explosive strength, coordination, and reactive ability. Previous studies have reported that jumping ability is associated with competitive level and is an important determinant of performance in rhythmic gymnasts [2,15]. Several previous studies have suggested that jumping performance reflects not only muscle strength and power but also neuromuscular function and the efficient use of the stretch–shortening cycle [8,9,10,11,12]. Thus, these physiological characteristics may be influenced by muscle quality, intracellular and extracellular fluid balance, and recovery status. Specifically, PhA has been associated with muscle strength and physical performance, including vertical jump performance in athletes [8,9,10]. In addition, PhA has been shown to reflect muscle quality and to be influenced by training load and recovery status [10,11,12]. Although PhA is not a direct measure of performance, it may reflect functional properties of muscle, such as muscle function and physiological condition, that underlie jumping performance. However, despite the potential value of PhA as a practical monitoring tool, its relationship with performance-related variables across different competitive levels in rhythmic gymnasts has not been sufficiently examined.

Therefore, this study aimed to examine the relationships among PhA, body composition, bone density, and jump performance (countermovement jump [CMJ] and reactive strength index [RSI]) in rhythmic gymnasts, stratified by competitive level, using a cross-sectional design. We hypothesized that PhA would be associated with skeletal muscle mass (SMM) and jump performance, and that these relationships would vary by competitive level.

## 2. Methods

### 2.1. Participants and Study Design

Participants were eligible for inclusion if they met the following criteria: (1) female rhythmic gymnasts aged 15–22 years, (2) active participation in national- or regional-level competitions, and (3) at least 10 years of competitive experience. Exclusion criteria were: (1) current musculoskeletal injury or illness that could affect performance testing and (2) inability to complete all measurements. As a result, 23 female senior-level rhythmic gymnasts (aged 15–22 years) were recruited. All participants had competitive experience at the national or regional level. Among them, five athletes who had won major domestic competitions in Japan were classified as the top group (17.0 ± 1.0 years). The top group consisted of the five regular members of a championship-level high school team, with no reserve gymnasts included. The remaining 18 athletes were classified as the general group (19.9 ± 1.1 years).

All measurements were performed on a single day during the competition season in Japan. The general group was assessed in August, whereas the top group was assessed in September. Both groups were evaluated during the competitive season when athletes were actively preparing for and participating in competitions. Athletes were asked to report their average weekly training duration over the previous 4 weeks. Training duration was obtained by self-report. Because all athletes in the top group belonged to the same team and followed the same training schedule, their reported training duration was identical. The participants underwent body composition, PhA, and bone density measurements under static conditions and were interviewed regarding their training hours over the previous 4 weeks. Subsequently, the participants performed a 15–20-min light warm-up, after which jump performance (CMJ and RSI) was evaluated. All measurements were performed by examiners from the same research team to minimize inter-examiner error, with sufficient familiarization regarding the measurement procedures.

All participants received prior written and verbal explanations regarding the purpose of the study and the measurement procedures, and written informed consent was obtained. This study was approved by the Keio University Research Ethics Committee (approval no.: 25-002; approved on 14 July 2025).

### 2.2. Measurement Items

#### 2.2.1. Body Composition and Phase Angle Measurements

The InBody M20 multifrequency BIA device (InBody Japan Co., Ltd., Tokyo, Japan) was used to measure body composition and PhA. Measurements were performed after the participants remained still in the supine position for at least 5 min and were instructed to empty their bladders prior to testing. Participants were barefoot during measurements, and electrodes were attached to two sites: the right wrist and right ankle. The body composition measurement parameters were body weight, SMM, percent body fat (PBF), body mass index (BMI), and PhA. In PhA, based on a previous study [12], the measurement frequency was set to 50 kHz, and PhA derived from R and Xc was recorded.

#### 2.2.2. Bone Density Measurement

Bone density was assessed using an FRS-100A ultrasonic calcaneal densitometer (Nippon Sigmax Co., Ltd., Tokyo, Japan). The participants were seated in a chair with the right calcaneus as the measurement site, and the speed of sound was used as the bone density index.

#### 2.2.3. Jumping Performance Measurement

To evaluate jumping performance, a jump measurement mat (S-CADE. Co., Ltd., Tokyo, Japan) was used to conduct the following measurements, as described in previous studies on gymnasts and athletes in aesthetic sports [15,16].

(1) Countermovement Jump (CMJ)

With both hands on the hips, the participants performed two maximal vertical jumps using a countermovement. Jump height (JH) was recorded for each trial, and the mean was used in the analyses.

(2) Reactive Strength Index (RSI)

Participants performed 10 consecutive rebound jumps with both hands on their hips. RSI was calculated as the jump height divided by the ground contact time (RSI = jump height/ground contact time). Two to three trials were conducted, and the average value was used for analyses.

### 2.3. Analysis

SPSS Statistics 30 (IBM, Armonk, NY, USA) was used for statistical analyses. Normality of the data was assessed using the Shapiro–Wilk test prior to the statistical analyses. No significant deviations from normality were detected in either group. An independent-samples *t*-test was used for comparisons of body composition, PhA, bone density, jump performance, and training time between the top and general groups. Homogeneity of variance was assessed using Levene’s test before conducting the independent-samples *t*-test. When the assumption of equal variances was violated, Welch’s *t*-test was used. Cohen’s d was calculated as the effect size, and 95% confidence intervals (CIs) were also calculated. Correlation analyses were conducted by competition level. Given the small sample size of the top group and the potential influence of outliers, Spearman’s rank correlation coefficient (ρ) was used to examine the relationships between PhA and body composition variables as well as jump performance (CMJ and RSI). For Spearman’s rank correlation coefficients, 95% CIs were estimated using bootstrap resampling with 5000 iterations to obtain stable CI estimates. Because the sample size of the top group was constrained by the availability of eligible elite athletes, an a priori sample size calculation was not feasible. Therefore, a post hoc sensitivity analysis was performed using G*Power version 3.1.9.7. The analysis indicated that, with a sample size of five, only very large correlations (|r| ≥ 0.88) could be detected with 80% power at a two-sided α level of 0.05. The significance level was set at 5% (*p* < 0.05).

## 3. Results

### 3.1. Characteristics of the Participants

Table 1 presents the participants’ body composition, bone density, PhA, jumping performance, and training time.

A comparison between the top (*n* = 5) and general (*n* = 18) groups revealed significant differences in bone density and training time (*p* = 0.041 and *p* < 0.001, respectively). Bone density was higher in the general group than in the top group, and training time was significantly longer in the top group. However, no statistically significant differences were observed in height, weight, BMI, SMM, PBF, PhA (50 kHz), CMJ, or RSI. Although these differences were not statistically significant, a large effect size was observed for PBF (d = −0.91), suggesting a lower body fat percentage in the top group. A moderate effect size was also observed for CMJ (d = 0.72), suggesting higher jump performance in the top group.

### 3.2. Relationships Between Variables

**Figure 1:**
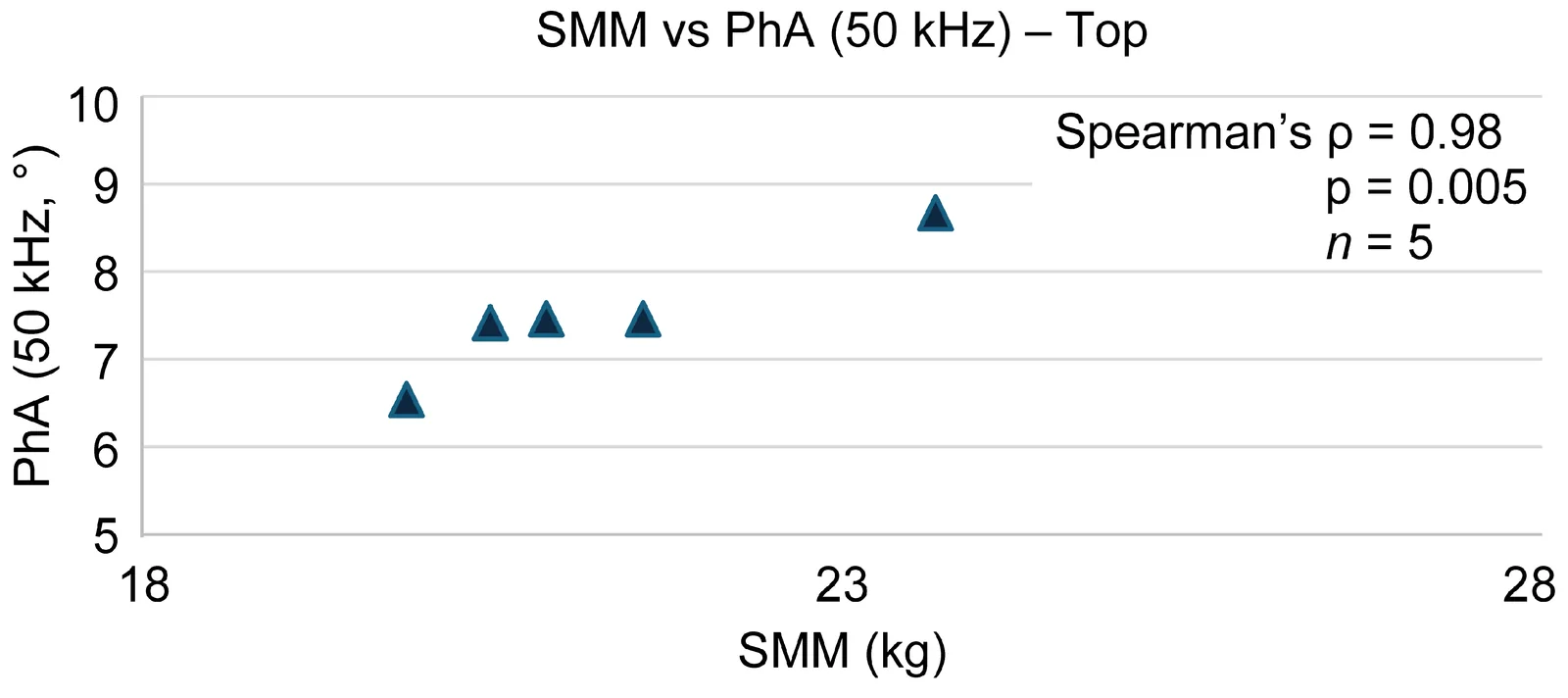
Relationship between SMM and PhA in the top group (*n* = 5). SMM, skeletal muscle mass; PhA, phase angle.

**Figure 2:**
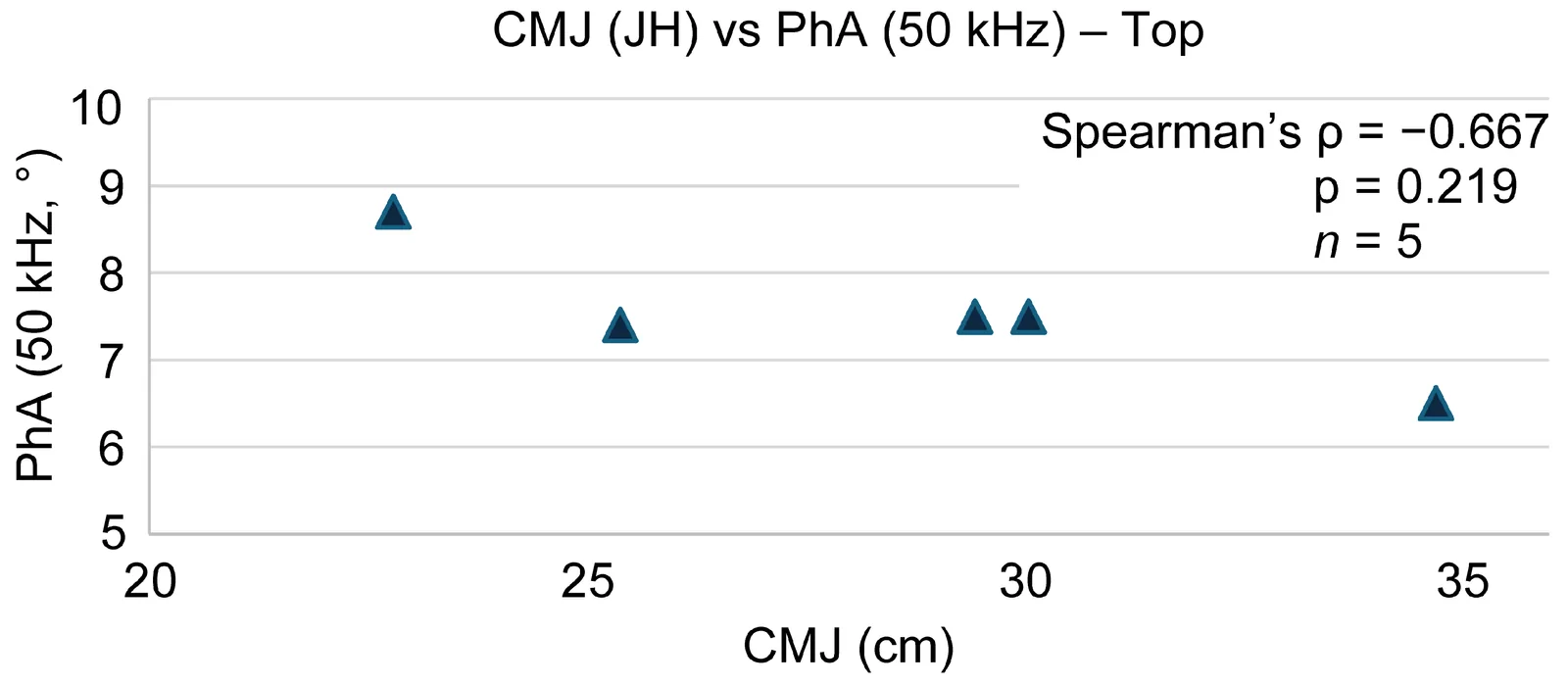
Relationship between CMJ and PhA in the top group (*n* = 5). CMJ, countermovement jump; PhA, phase angle.

**Figure 3:**
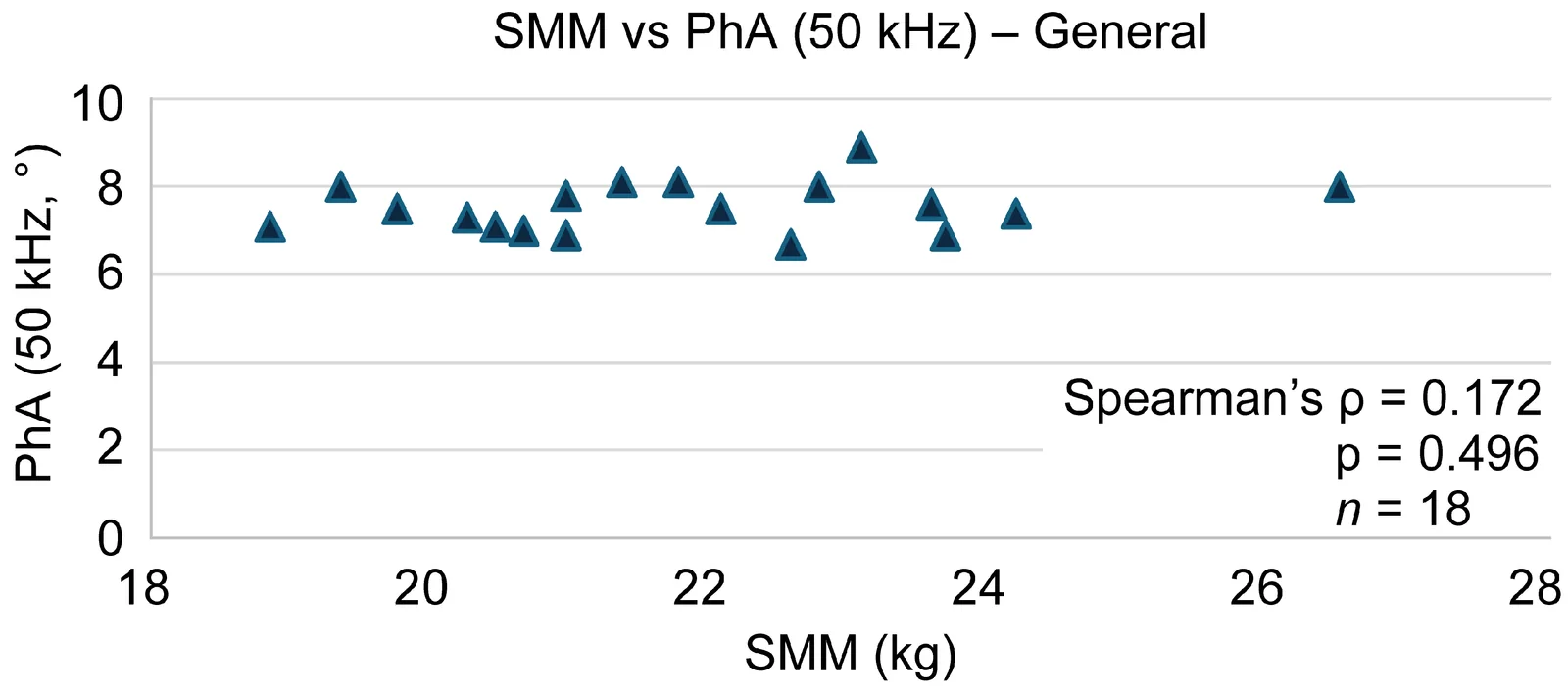
Relationship between SMM and PhA in the general group (*n* = 18). SMM, skeletal muscle mass; PhA, phase angle.

**Figure 4:**
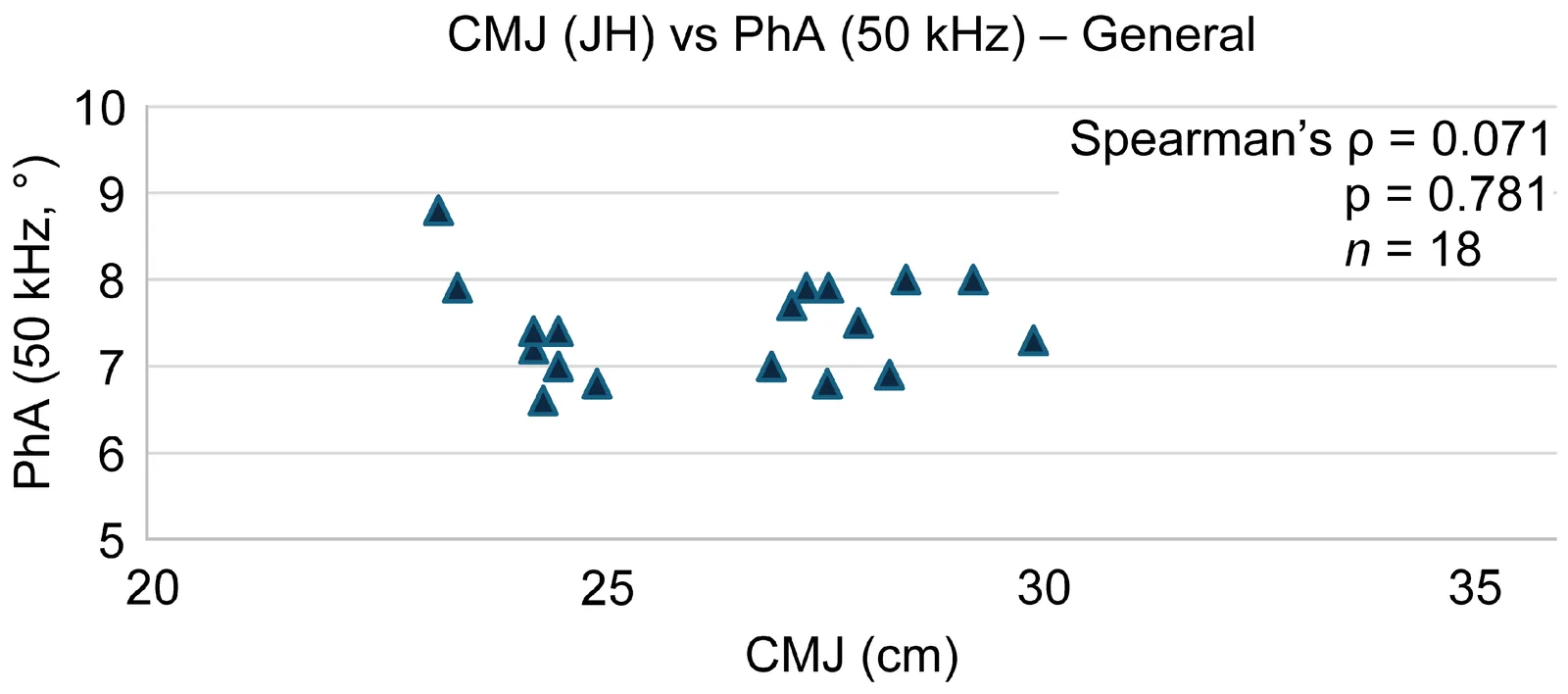
Relationship between CMJ and PhA in the general group (*n* = 18). CMJ, countermovement jump; PhA, phase angle.

In the top group (Table 2), SMM showed strong positive correlations with PhA at 50 kHz (ρ = 0.98, *p* < 0.01). In contrast, CMJ showed a moderate negative correlation with PhA (ρ = −0.67); however, this correlation was not statistically significant. In the general group (Table 3), no clear relationships were observed between PhA and body composition variables or jump performance. Figure 1 and Figure 2 illustrate the relationships between SMM and PhA and between CMJ and PhA in the top group, whereas Figure 3 and Figure 4 show the corresponding relationships in the general group for comparison.

## 4. Discussion

In this study, we analyzed the relationships among body composition, PhA, bone density, and jumping performance in senior rhythmic gymnasts classified by their competitive achievements. However, because the top group comprised only five athletes, the analysis was conducted as an exploratory cross-sectional study. While differences in mean values between groups were limited, the relationships between PhA and performance-related variables differed according to competitive level. These results partially supported our hypothesis. Specifically, associations between PhA, SMM, and CMJ were observed only in the top group. However, the association with jumping performance differed from our expectations.

PhA reflects an indicator of the capacitance of the cell membrane and distribution of water inside and outside the cell; in athletes, relationships between PhA and muscle function and physical condition have been reported [6,7,8,13]. Although differences exist by age and sport-specific characteristics, PhA in female athletes generally falls within approximately 6–8° [17]. Furthermore, among rhythmic gymnasts, the PhA at the sub-elite level has been reported to be 6.7 ± 0.5° [1]. The PhA values of the participants in this study (approximately 7.4–7.5°) fell within this range, consistent with previous reports.

The strong positive correlation observed between PhA and SMM in the top group is consistent with previous studies reporting a positive association between PhA and SMM in athletes [6,7,8,13]. Maintaining SMM is particularly important for athletic performance, and the observed association may reflect the close relationship between PhA and SMM in highly trained athletes. This relationship may also have been influenced by the relatively homogeneous characteristics of the athletes in the top group. However, given the very small sample size of the top group, this finding should also be interpreted as exploratory and requires confirmation in a larger study to elucidate the mechanism. However, an association may exist between higher CMJ performance and lower PhA in the top group. Interestingly, this observation differed from previous reports demonstrating positive associations between PhA and muscle strength or power [6,8]. Generally, higher PhA values indicate better cell membrane integrity and optimal intracellular and extracellular water balance. In this study, although no clear difference in SMM was observed between the top and general groups, CMJ was higher in the top group, and PBF tended to be lower. These findings suggest that differences in performance may not be explained solely by SMM. Although rhythmic gymnastics routines are brief, the sport involves extremely high exercise intensity [18,19]. Previous studies have demonstrated that elite rhythmic gymnasts are exposed to high training loads during the competitive season and that appropriate monitoring of training load and recovery is important for maintaining performance [20,21]. In addition, recent evidence suggests that although PhA generally increases with greater muscle mass, it may transiently decrease during periods of high training load or functional overreaching, reflecting accumulated physiological stress rather than reductions in muscle mass [11]. Thus, because the present measurements were performed during the competitive season, the top gymnasts may have maintained high CMJ performance despite accumulated training-related fatigue. One possible explanation is that the lower PhA observed in athletes with higher CMJ performance may reflect the physiological demands associated with training and competition during the competitive season rather than poorer muscle status. However, because variables such as internal load, recovery status, hydration, and menstrual function were not directly assessed in this study, the mechanisms underlying this association remain unclear, and any physiological interpretation remains hypothetical. Accordingly, the observed association between CMJ performance and PhA in the top group should be interpreted with caution. This interpretation requires confirmation in larger longitudinal study.

This study identified group differences in bone density and training duration, whereas no clear differences were observed in the other variables. However, the groups differed in age, and age-related differences in skeletal maturation may have contributed to the observed difference in bone density. In the top group, bone density tended to be positively associated with jump performance (CMJ and RSI) but negatively associated with PhA. Although the physiological significance of these findings remains unclear, PhA has been suggested to be influenced by training load and recovery status [11], whereas bone density primarily reflects skeletal adaptation to mechanical loading. Therefore, these indices may not necessarily change in parallel. These findings require confirmation in longitudinal studies, particularly those that track athletes throughout growth and maturation.

The present study has several limitations. First, the top group consisted of only five athletes from a single Japanese high-school team, limiting the statistical power and generalizability of the findings. A post hoc sensitivity analysis indicated that, with a sample size of five, only very large correlations (|r| ≥ 0.88) could be detected with 80% power at a two-sided α level of 0.05. Therefore, the correlation analyses in the top group should be regarded as exploratory, and the observed associations may have been substantially influenced by individual observations. In addition, the results of normality tests should be interpreted with caution because such tests have limited informativeness in very small samples. Furthermore, because multiple correlation analyses were performed without adjustment for multiple comparisons, the possibility of inflated Type I error cannot be excluded. Second, the cross-sectional design precludes causal inferences regarding the relationships between PhA and performance-related variables. Moreover, all measurements were performed on a single day during the competitive season, making it impossible to evaluate how PhA changes across different phases of the training and competition season or in response to accumulated training load over time. The lack of direct assessments of internal load (e.g., session rating of perceived exertion) further limits the interpretation of the observed associations. Finally, several factors known to influence PhA measurements were not controlled, including hydration status, fatigue, recovery status, menstrual status, and other day-to-day physiological variations. Bone health was assessed using calcaneal ultrasound rather than dual-energy X-ray absorptiometry, and factors related to bone health and RED-S, including energy availability, dietary intake, and fracture history, were not evaluated either. These uncontrolled factors should be considered when interpreting the present findings. Taken together, the present findings should be regarded as hypothesis-generating rather than confirmatory. Future studies using larger sample sizes and longitudinal designs are needed to clarify the relationships between PhA, performance-related variables, and physiological condition in rhythmic gymnasts.

## 5. Conclusions

This study explored the relationships between body composition, PhA (50 kHz), bone density, and jumping performance in rhythmic gymnasts stratified by competitive level. Although group differences in mean values were limited, PhA was positively associated with SMM in the top group, whereas a possible association with CMJ performance was observed and should be interpreted cautiously. These exploratory findings suggest that the associations between PhA and performance-related variables may differ by competitive level; however, the potential influence of age and maturation cannot be ruled out. Given the exploratory nature of the study and the very small sample size of the top group, these findings should be considered preliminary and require confirmation in larger and longitudinal studies.

## Figures and Tables

**Table 1:** Physical characteristics and jump performance of the participants.

Variable	Unit	Top (*n* = 5)	General (*n* = 18)	*p*	d	95% CI
Height	cm	158.0 ± 3.24	159.05 ± 4.24	0.614	−0.26	−1.24 to 0.74
Weight	kg	47.32 ± 3.86	50.93 ± 4.80	0.138	−0.78	−1.77 to 0.27
BMI	kg/m^2^	19.00 ± 2.22	20.11 ± 1.48	0.195	−0.68	−1.66 to 0.36
SMM	kg	21.32 ± 1.46	21.85 ± 1.92	0.572	−0.29	−1.27 to 0.71
PBF	%	16.78 ± 6.76	20.86 ± 3.75	0.086	−0.91	−1.91 to 0.15
Bone density	m/s	1535.20 ± 13.98	1558.83 ± 22.83	0.041 *	−1.10	−2.11 to −0.02
PhA (50 kHz)	°	7.52 ± 0.78	7.45 ± 0.56	0.824	0.11	−0.88 to 1.10
CMJ	cm	28.46 ± 4.57	26.47 ± 2.13	0.170	0.72	−0.32 to 1.71
RSI	m/s	2.07 ± 0.27	2.05 ± 0.23	0.885	0.08	−0.90 to 1.08
Training time	h	28.0 ± 0	19.3 ± 8.8	<0.001 *	1.10	0.01 to 2.10

Values are presented as mean ± standard deviation (SD). Cohen’s d is reported with its 95% confidence interval (CI). BMI, body mass index; SMM, skeletal muscle mass; PBF, percent body fat; PhA, phase angle; CMJ, countermovement jump; RSI, reactive strength index. * *p* < 0.05.

**Table 2:** Correlations between PhA and body composition/jumping performance in the top group (*n* = 5).

Variable	PhA 50 kHz (ρ)	95% CI	*p*
SMM	0.98	0.75 to 1.00	<0.01
PBF	0.05	−1.00 to 1.00	0.94
CMJ	−0.67	−1.00 to 0.92	0.22

Note: Spearman’s rank correlation coefficients (ρ) are presented. The 95% confidence intervals (CIs) were estimated using bootstrap resampling (5000 iterations). SMM, skeletal muscle mass; PBF, percent body fat; CMJ, countermovement jump.

**Table 3:** Correlations between PhA and body composition/jumping performance in the general group (*n* = 18).

Variable	PhA 50 kHz (ρ)	95% CI	*p*
SMM	0.17	−0.28 to 0.57	0.50
PBF	0.26	−0.18 to 0.63	0.30
CMJ	0.07	−0.45 to 0.61	0.78

Note: Spearman’s rank correlation coefficients (ρ) are presented. The 95% confidence intervals (CIs) were estimated using bootstrap resampling (5000 iterations). SMM, skeletal muscle mass; PBF, percent body fat; CMJ, countermovement jump.

## Data Availability

The data that support the findings of this study are available from the corresponding author upon reasonable request due to privacy.

## Abbreviations

The following abbreviations are used in this manuscript:

BIA	bioelectrical impedance analysis
BMI	body mass index
CMJ	countermovement jump
PBF	percent body fat
PhA	phase angle
R	resistance
RSI	reactive strength index
SMM	skeletal muscle mass
Xc	reactance
